# Smallpox

**Published:** 1899-02

**Authors:** 


SMALLPOX.—The very extensive prevalence of smallpox throughout Europe and America at the present time may, in a large measure, be ascribed to the senseless outcry against vaccination, which, breaking out afresh in England some years ago, has gained such headway as to lead to a repeal virtually of the vaccination act. Any one now can avoid vaccination or the vaccination of his children by simply saying, "I have conscientious scruples against it." It has spread to this country, and the prejudice is much more widespread than most persons would suppose. This is most remarkable, in the face of the demonstrated fact that at one time smallpox was nearly eradicated—by vaccination—from civilized countries, there having been, for instance, only one death from smallpox in London in the year 1890. The crusade against this great blessing seems to be a persistence of the old superstitious notions of the past, that to prevent disease is contravening the will of God. Medical scholars all know the history of the great outbreak of smallpox in Montreal only a few years ago, when so many resorted to prayers, processions and holy water to ward off the scourge, refusing vaccination, and how they perished by the scores.

The JOURNAL would admonish its readers to be on guard, for the disease is very prevalent, and no one knows when he will be called on to treat it. Every physician should personally urge the people in his community to be vaccinated and revaccinated. Every child should be promptly vaccinated as a condition to being permitted to go to school. In many cities this is enforced. In Texas, like all other measures for public protection, it is neglected. In this issue we have devoted considerable space to items on smallpox, because of the imminent danger of its spread. The following data from the Supplementary Health Reports, Marine Hospital Service, January 6, 1899, is timely and of interest:

"In 1871 Germany, with a population of 50,000,000, lost 143,000 lives by smallpox. In 1874 vaccination was made obligatory, and the result has been such a rapid and signal reduction of mortality that today only 116 victims are annually sacrificed to the disease.

"The other side of the shield is strikingly depicted by Prof. Osler on page 69 of his Practice of Medicine (second edition), where he describes the dreadful ravages of smallpox amongst the unvaccinated population of Montreal, where, in the ten months following February 28, 1885, thousands were stricken with smallpox and 3164 died. At that time one of the directors of our biological department resided in northern New York, and he recalls vividly the consternation that prevailed in all sections contiguous to the pest-ridden city."

Along with progress in other matters, advancement has been made in the *methods* of vaccination. A perfectly pure, antiseptic lymph, put up in sealed tubes, can now be had from Parke, Davis & Co., with instructions how to use, and with literature giving statistics on vaccination.

